# Saccharomyces boulardii alleviates ulcerative colitis carcinogenesis in mice by reducing TNF-α and IL-6 levels and functions and by rebalancing intestinal microbiota

**DOI:** 10.1186/s12866-019-1610-8

**Published:** 2019-11-06

**Authors:** Chunsaier Wang, Wenbin Li, Hongying Wang, Yiming Ma, Xinhua Zhao, Xudong Zhang, Hong Yang, Jiaming Qian, Jingnan Li

**Affiliations:** 1 Department of Gastroenterology, Peking Union Medical College Hospital, Chinese Academy of Medical Sciences and Peking Union Medical College, Beijing, 100730 China; 2 National Cancer Center/Cancer Hospital, Chinese Academy of Medical Sciences and Peking Union Medical College, Beijing, 100021 China; 3 School of Biomedical Sciences and Pharmacy, University of Newcastle, Newcastle, New Lambton Heights, New South Wales, Australia

**Keywords:** Ulcerative colitis carcinogenesis, TNF-α, IL-6, *Saccharomyces boulardii*, Intestinal microbiota

## Abstract

**Background and Aims:**

To explore the inhibition mechanism of *Saccharomyces boulardii* (*S. boulardii*) on ulcerative colitis (UC) carcinogenesis.

**Methods:**

C57BL/6 mice were treated with azoxymethane and dextran sulfate sodium (AOM/DSS) to develop a UC carcinogenesis model. The treatment group was lavaged with *S. boulardii* (5 × 10^7^ CFU/d) for 12 weeks. The mice were sacrificed and the tumor load in the treatment group was compared with that of a control group. The levels of TNF-α and IL-6 in colon tissue were measured by enzyme-linked immunosorbent assays. The influence of *S. boulardii* on TNF-α and IL-6 regulation was also investigated using different colon cell lines. Differences in intestinal microbiota in both stool and intestinal mucosa samples were assessed using 16S rDNA sequencing.

**Results:**

*S. boulardii* treatment reduced AOM/DSS-induced UC carcinogenesis in mice, as indicated by the reduced tumor load and reduced TNF-α and IL-6 levels in vivo, as well its effects on TNF-α and IL-6 activities in vitro. Significant changes in both fecal and mucosal microbiota were observed among the control, the AOM/DSS treated, and AOM/DSS plus *S. boulardii* treated groups. For fecal microbiota, the AOM/DSS treated group was lower in *Lactobacillus*, but higher in *Oscillibacter* and *Lachnoclostridium* than the control group. After intervention with *S. boulardii*, the percentage of *Bacillus* and *Lactococcus* increased, but *Lachnoclostridium*, *Oscillibacter, Bacteroides,* and *Pseudomonas* decreased. For the intestinal mucosal microbiota, the AOM/DSS treated group was lower in *Bifidobacterium* and *Ruminococcaceae_UCG-014* and higher in *Alloprevotella* than the control group. After *S. boulardii* exposure, the percentage contributions of *Lachnoclostridium* and *Lachnospiraceae_NK4A136* increased.

**Conclusions:**

*S. boulardii* effectively reduced UC carcinogenesis in an AOM/DSS induced mice model. This positive result can likely be attributed to the reduction of TNF-α and IL-6 levels or the blockade of their function combined with alterations to the intestinal microbiota.

## Background

Ulcerative colitis (UC) is a chronic inflammatory disease of the colon [1]. Individuals with UC are at increased risk of developing colorectal cancer over healthy individuals [2]. One possible cause of UC carcinogenesis is repeated cycles of epithelial cell injury and repair [3]. During this process, cells are immersed in a chronic inflammatory cytokine milieu, with the overproduction of pro-inflammatory cytokines such as tumor necrosis factor alpha (TNF-α) and interleukin 6 (IL-6). TNF-α is involved in all stages of carcinogenesis, such as cellular transformation, proliferation, angiogenesis, and metastasis [4]. IL-6 is another key cytokine that plays an important role in cancer progression [5]. Its pro-tumorigenic influence is largely mediated by the signal transducer and activator of transcription 3.

An inflammatory environment is fundamental in the initial stages of UC carcinogenesis pathogenesis [6]. In mice, it has been shown that dextran sulfate sodium (DSS), an agent with direct toxic effects on the colonic epithelium, can cause a chronic inflammatory state and has been used for the induction of colitis in mice [7, 8]. Indeed, prolonged exposure to DSS can induce tumors in mice. The tumor development is hastened by pretreatment with the genotoxic agent azoxymethane (AOM).

An increasing body of evidence has shown that intestinal microbiota can influence the occurrence of UC [9], because of its induction of persistent intestinal inflammation. In recent years, probiotics have been used as supplements in UC patients, to help maintain a healthy microbiota to control UC remission and UC carcinogenesis. The probiotic *S. boulardii* has shown high efficacy in the treatment of UC [10]. But the possible role of *S. boulardii* in the prevention of UC carcinogenesis and the underlying mechanisms have not been investigated thus far. In the current study, we explored the curative influence of *S. boulardii* and the mechanisms of same on UC carcinogenesis. Furthermore, variations in fecal and mucosa microbiota, which have been reported to have different bacterial compositions [11], and their possible association with UC carcinogenesis, were also assessed. Our results indicate that *S. boulardii* supplementation could alleviate UC carcinogenesis in mice through reducing levels of TNF-α and IL-6 or impairing their function and altering the intestinal microbiota. This study sheds new light on the molecular mechanism of UC carcinogenesis.

## Methods

### Animals, cell lines and bacterial strains

Male C57BL/6 mice (*n* = 50, 6–8 weeks old) were purchased from Vital River Laboratory Animal Technology Co. Ltd. (Beijing, China). All animals were housed in controlled conditions (temperature 22 ± 1 °C, humidity 40–60% and 12 h dark/light cycle). The mice had free access to a standard laboratory diet and water for one week for acclimatization.

Human colorectal adenocarcinoma cell lines, HCT-116 and Caco-2, were obtained from the laboratory of Wang at the National Cancer Center/Cancer Hospital of the Chinese Academy of Medical Sciences and Peking Union Medical College (Beijing, China), and the human embryonic intestinal mucosa derived cell line CCC-HIE-2 was obtained from the Basic Research Institute of Peking Union Medical College (Beijing, China). HCT-116 was cultured in Iscove’s modified Dulbecco’s medium (IMDM; Thermo Fisher Scientific, USA) supplemented with 10% fetal bovine serum (FBS; Hyclone, USA). Caco-2 was cultured in minimum Eagle’s medium (MEM; Thermo Fisher Scientific, USA) supplemented with 10% FBS and 1% non-essential amino acids (NEAA; Thermo Fisher Scientific, USA). CCC-HIE-2 was cultured in Dulbecco’s modified eagle medium-high glucose (DMEM-H; Thermo Fisher Scientific, USA) supplemented with 20% FBS (Hyclone, USA) and 1% NEAA (Thermo Fisher Scientific, USA). To each of these media, both 100 mg/mL streptomycin and 100 U/mL penicillin were added. All cells were cultured at 37 °C in a humidified atmosphere with 5% CO_2_.Cells were trypsinized and subcultured every 2–3 days and were only used for up to 20 passages.

*S. boulardii* was purchased from China Medical System Holdings Ltd. (Beijing, China) and its culture medium, malt extract medium (MEM), was purchased from BeNa Culture Collection (Suzhou, China). A single colony was inoculated into 10 mL of MEM (pH 6.2) and cultured overnight at 30 °C with constant shaking. Cells in their logarithmic growth phase (12–16 h culture) were selected and used for all experiments.

### Development of a UC carcinogenesis model and treatment procedures

Fifty mice were randomly divided into three groups. (1) AOM/DSS treated group (+AOM/DSS): 20 mice were given a single intraperitoneal injection of AOM (12.5 mg/kg body weight in normal saline, Sigma-Aldrich). One week later, the mice were given 2.5% DSS (Mpbio, Solon, OH, USA) added to the drinking water for 5 days, which was then replaced with drinking water; (2) AOM/DSS plus *S. boulardii* group (+AOM/DSS + *Sb*): 20 mice were first given the same amount of AOM and DSS, then they were lavaged with *S. boulardii* (5 × 10^7^ colony forming unit (CFU)/d) from the day of the AOM injection for 12 weeks (5 days/week); (3) Control group: 10 mice with no AOM/DSS or *S. boulardii* treatment were used as the control group.

### Specimen processing

At the end of the 12th week, mice were anesthetized with diethyl ether and sacrificed via transcardiac perfusion and colon tissues were removed. The colons were slit open longitudinally along the main axis and washed with normal saline (0.9% NaCl). The diameter of each tumor was measured using a sliding caliper, and the total tumor load of each colon was calculated, where the load was the sum of the tumor diameters. Subsequently, the entire colon was divided into four sections. The section near the anus was washed two times vigorously with normal saline to remove non-adherent bacteria. This section was then flash-frozen in liquid nitrogen and stored at − 80 °C for subsequent microbiota analysis. The remaining sections were used for enzyme-linked immunosorbent assay (ELISA) analysis and histopathological examination.

### Enzyme-linked Immunosorbent assay (ELISA) for TNF-α and IL-6

The levels of TNF-α and IL-6 in the colon mucosa were measured using commercial mouse TNF-α and IL-6 ELISA kits (eBioscience, USA), according to the manufacturer’s instructions. The absorbance of the final products was measured on a microplate reader at a wavelength of 450 nm. The results were expressed as pg/mg tissue. Eight mice were randomly selected from each group for ELISA assays.

### Histopathological examination of tumor tissues

The colon tissue was first fixed in 10% buffered formalin, then dehydrated, and paraffin embedded. Four micrometer sections were cut with a microtome for hematoxylin-eosin (HE) staining. After staining, sections were dehydrated through an increasing concentration series of ethanol and xylene.

### Luciferase reporter assays

Caco-2, HCT-116, and CCC-HIE-2 cells were split and seeded into separate 24-well plates at a density of 1 × 10^5^ cells per cm^2^. Cells were then incubated overnight under standard culture conditions and were then transfected with plasmid constructs expressing TOP-Flash targeting LEF1/TCF-4-3’UTR (400 ng/well; Millipore, USA). Co-transfection with pRL-TK plasmid (Promega, USA) was used as an internal control to test the efficacy of transient transfection (40 ng/well). Transfection was carried out following the manufacturer’s protocol in serum-free medium. Cells were fed with fresh complete culture medium 5 h after the transfection. After 24 h, the monolayer of cells was rinsed with serum-free medium and stimulated by TNF-α (50 ng/mL; Peprotech) or IL-6 (50 ng/mL; Peprotech) for 2 h. The positive reporter plasmid has TCF/LEF consensus binding sites that drive expression of firefly luciferase, and the negative reporter lacks TCF/LEF binding sites. So the luciferase activity represents the transcription activity of TCF/LEF.

For the co-culture experiment, transfection was performed first as described above. After 24 h culture, confluent cells were rinsed with serum-free medium. *S. boulardii* cells were washed twice with phosphate-buffered saline (PBS, pH = 7.4) and mixed with cultured cells at a final concentration of 1 × 10^5^ CFU/mL, which was the same concentration of the cultured cells. The multiplicity of infection (MOI) is about 1. The mixture was cultured for 4 h and then stimulated by TNF-α (50 ng/mL) or IL-6 (50 ng/mL) for 2 h. The activity of luciferase was measured using the dual reporter gene system (Promega, USA) with an automated chemiluminescence detector (BioTek, Germany). The transfections were repeated at least twice to ensure reproducibility of the results.

### Microbiota analysis

Stool samples were collected just before AOM injection and before sacrifice. Stools from six mice were randomly selected from each group. Stool samples and intestinal mucosa samples were sent to Allwegene (Beijing, China) to analyze the differences in intestinal microbiota using16S rDNA sequencing.

Microbial genomic DNA was isolated using a QIAamp DNA Micro Kit according to the manufacturer’s instructions. The purity and quality of the genomic DNA were examined using 0.8% agarose gels. The final quantity and quality of the DNA were assessed at 260 and 280 nm using an ultraviolet spectrophotometer and stored at − 20 °C before further analysis. The V3-V4 hypervariable regions of the 16S rDNA gene were amplified using forward primer 338F (ACTCCTACGGGAGGCAGCAG) and reverse primer 806R (GGACTACHVGGGTWTCTAAT). For each sample, a 10-digit barcode sequence was added to the 5′ end of the forward and reverse primers. The PCR was carried out on a Mastercycler Gradient (Eppendorf, Germany) using 25 μl reaction volumes, containing 12.5 μl 2× Taq PCR MasterMix, 3 μl BSA (2 ng/μl), 2 primer (5 uM), 2 μl template DNA, and 5.5 μl ddH_2_O. Cycling parameters were 95 °C for 5 min, followed by 32 cycles of 95 °C for 45 s, 55 °C for 50 s and 72 °C for 45 s with a final extension at 72 °C for 10 min. Three PCR products per sample were pooled to mitigate reaction-level PCR biases. The PCR products were purified using a QIAquick Gel Extraction Kit (QIAGEN, Germany), quantified using Real Time PCR, and sequenced by Allwegene using the Illumina Miseq PE300 sequencing platform (Illumina Inc., CA, USA).

The raw sequencing data were first screened and sequences that were shorter than 200 bp, had a low quality score (≤ 20), contained ambiguous bases or did not exactly match to primer sequences and barcode tags were removed from further consideration. Qualified reads were separated using the sample-specific barcode sequences and trimmed with Illumina Analysis Pipeline software (version 2.6). The datasets were analyzed using QIIME. The sequences were clustered into operational taxonomic units (OTUs) at a similarity level of 97%, to generate rarefaction curves and to calculate the richness and diversity indices. The Ribosomal Database Project (RDP) Classifier tool was used to classify all sequences into different taxonomic groups. To examine the similarity between different samples, clustering analyses and PCA were used based on the OTU information from each sample using the R software. The evolution distances between microbial communities from each sample were calculated using the tayc coefficient and represented as an unweighted pair group method with arithmetic mean (UPGMA) clustering tree describing the dissimilarity (1-similarity) between multiple samples. A Newick-formatted tree file was generated through this analysis. To compare the membership and structure of communities in different samples, heat maps were generated with the top 20 OTUs using Mothur.

### Statistical analysis

Data are presented as the mean ± SE (standard error). All statistical analyses were performed using GraphPad Prism Software Version 6.0 (GraphPad Software Inc., La Jolla, CA, USA). Statistical differences between experimental variants were assessed by two-tailed independent t-tests and data from more than three groups were analyzed by one-way ANOVA. Anosim and metastats were used for microbiota analysis. A *p*-value of less than 0.05 was considered statistically significant.

## Results

### Establishment of the UC carcinogenesis mice model through AOM/DSS treatment

**Fig. 1:**
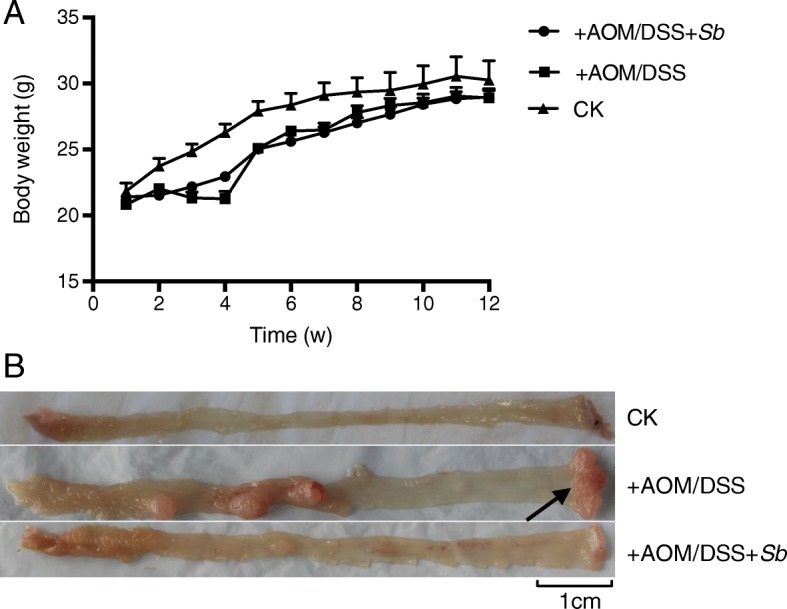
**a**: Changes of body weight over the course of the experiment. **b**: Colon tissue showing colonic tumors in the AOM/DSS treated group (+AOM/DSS) and the AOM/DSS plus *S. boulardii* treated group (+AOM/DSS + *Sb)*, none were observed in the control group (CK, no AOM/DSS or *S. boulardii* treatment)

C57BL/6 mice were treated with AOM/DSS to establish a UC carcinogenesis mice model. First, we examined the general health of the mice, with or without AOM/DSS treatment, or *S. boulardii* exposure. As shown in Fig. 1a, the body weight of all three groups, including the control group (CK, no AOM/DSS or *S. boulardii* treatment), the AOM/DSS treated group (+AOM/DSS), and the AOM/DSS plus *S. boulardii* treated group (+AOM/DSS + *Sb*), had increased by about 40% at the end of the 12 weeks. No significant difference in body weight was observed among the three groups at this stage. However, significant body weight loss was observed in the AOM/DSS treated group on the third day of DSS treatment (day 10 of the experiment), but this lost weight was gradually recovered when DSS was replaced with normal drinking water on day 21 of the experiment. Weight loss was also observed in the AOM/DSS + *Sb* group, but the loss was not as significant as the AOM/DSS treated group. No apparent weight loss was observed in the control mice. In addition to the weight loss, loose and bloody stools and other colitis symptoms were observed both in the AOM/DSS treated group and the AOM/DSS + *Sb* group, over the same period as the weight loss. During the ninth week, some of the mice in both treatment groups were observed to have bloody stools again, with anal prolapse on the tenth week; the bloody stools and anal prolapse occurrences were more severe in the AOM/DSS treated group than in the AOM/DSS + *Sb* group.

**Fig. 2:**
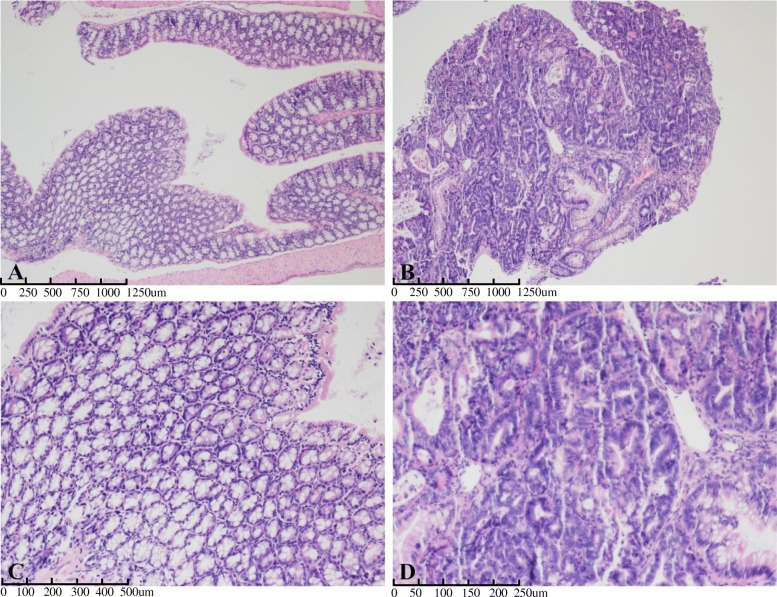
Representative images of hematoxylin and eosin staining of colon tissue examined under a microscope. **a**: Normal control group: colonic mucosal glands were normal, and the structure was regular, 40×. **b**: Model control group: colonic gland structural disorders, deep nuclear staining, and cytoplasm-to-nuclear ratio decreased, 40×. **c**: Normal control group, 100×. **d**: Model control group, 200×

The mice were sacrificed at the end of the 12th week. Examination of the colon tissues showed visible colorectal tumors in both the AOM/DSS treated mice and the AOM/DSS + *Sb* treated mice; no tumors were observed in the control mice (Fig. 1b). Tumors were mainly located in the distal portion of the colon. Tumor fusion and ring growth at the end of the rectum were frequently observed in the tissues of mice that had suffered anal prolapse (Fig. 1b, arrow). HE staining of the colon tissues showed a normal and typical structure in the control group, but the AOM/DSS treated tissue showed darker HE staining, enlarged nuclei (or decreased ratio of cytoplasm to nucleus), and colonic gland structure disorder (Fig. 2). This further confirms that UC carcinogenesis was induced by AOM/DSS treatment.

### S. Boulardii treatment reduces AOM/DSS induced UC carcinogenesis in mice

**Fig. 3:**
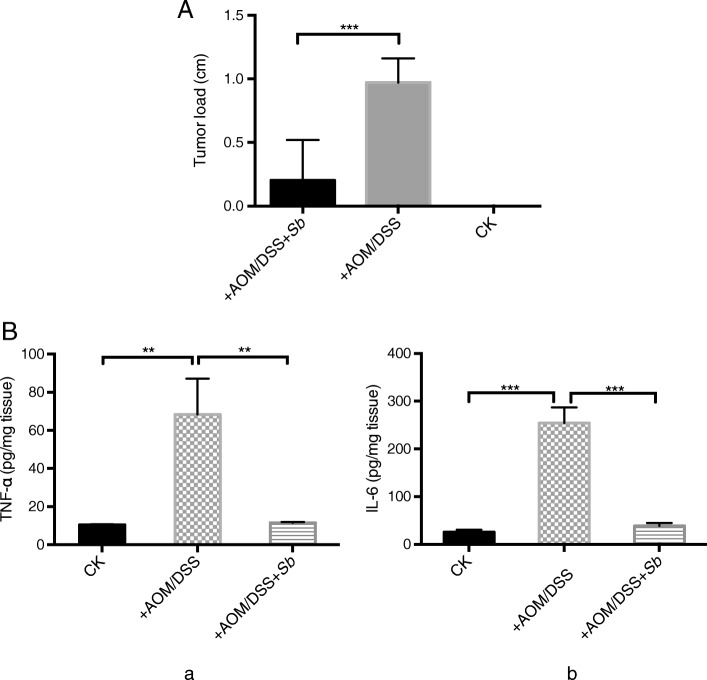
**a**: Comparison of mean tumor loads (sum of tumor diameters) of different groups. **b**: Levels of colonic TNF-α (a) and IL-6 (b) with or without treatments of AOM/DSS and *S. boulardii*

Treatment with AOM and DSS led to 100% incidence of colonic neoplasms in mice. *S. boulardii* administration reduced the incidence of colonic neoplasms to 63.16%. Moreover, the mean tumor load (sum of the tumor diameters) was reduced from 0.97 ± 0.19 cm to 0.20 ± 0.07 cm (*p* = 0.0006; Fig. 3a). No colonic tumors were observed in the control group mice.

### S. Boulardii treatment reduces TNF-α and IL-6 levels in AOM/DSS treated mice

To further examine the effects of *S. boulardii* on UC carcinogenesis, the levels of TNF-α and IL-6 in colon tissues were measured using ELISA. As illustrated in Fig. 3b, the levels of colonic tissue TNF-α (*p* < 0.01) and IL-6 (*p* < 0.001) were significantly higher in the AOM/DSS treated group than that in control group. The levels of TNF-α and IL-6 in +AOM/DSS + *Sb* treated mice were low, similar to the control group. This suggests that *S. Boulardii* treatment could reduce the high levels of TNF-α and IL-6 induced by AOM/DSS.

### S. Boulardii treatment reduces the effects of TNF-α and IL-6 in different cell lines

**Fig. 4:**
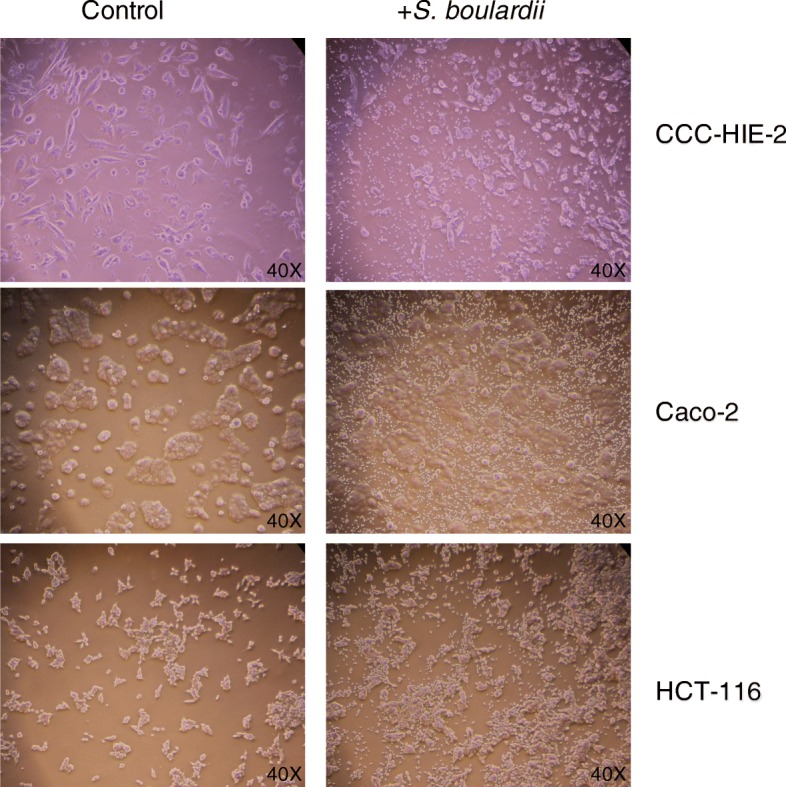
Phase contract microscopy images of cell line co-culture with *S. boulardii* (40×)

**Fig. 5:**
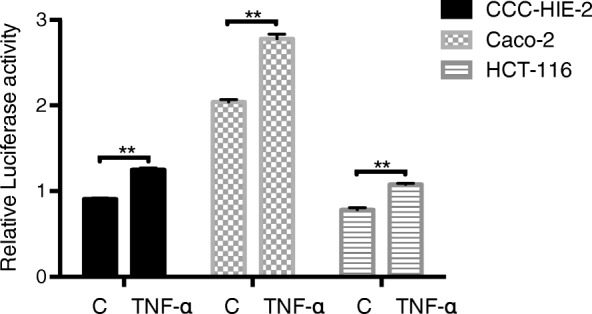
Relative luciferase activity with or without TNF-α treatment. C: control; TNF-α: treated with 50 ng/mL TNF-α for 2 h

**Fig. 6:**
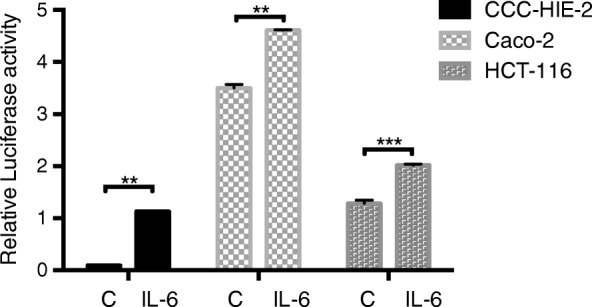
Relative luciferase with or without IL-6 treatment. C: control; IL-6: treated with 50 ng/mL IL-6 for 2 h

**Fig. 7:**
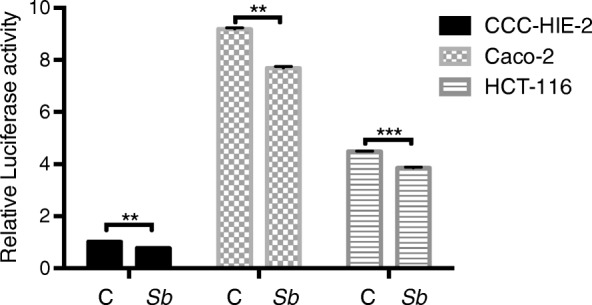
Co-culture of cells with *S. Boulardii* reduces the relative luciferase activity stimulated by TNF-α. C: control (cells treated with TNF-α but not *S. boulardii*); Sb: co-cultured with *S. boulardii*

**Fig. 8:**
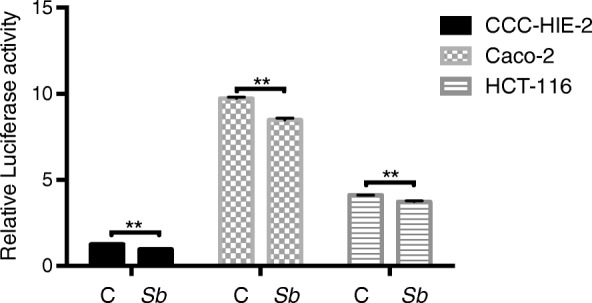
Co-culture of cells with *S. Boulardii* reduces the relative luciferase activity stimulated by IL-6. C: control (cells treated with IL-6 but not *S. boulardii*); Sb: co-cultured with *S. boulardii*

Three different cell lines (CCC-HIE-2, Caco-2, and HCT-116) were used to further investigate the possible mechanisms underpinning the effects of *S. boulardii* on UC carcinogenesis. Microscopic images showed that *S. boulardii* cells accumulated around the cell peripheries, but the cell morphologies were not altered (Fig. 4). Addition of TNF-α or IL-6 to the cell culture significantly increased the luciferase activity (Figs. 5 and 6). Co-culture of the cell lines with *S. boulardii* (1 × 10^5^ CFU/mL) significantly reduced the relative luciferase activity stimulated by TNF-α (Fig. 7) or IL-6 (Fig. 8). These results suggest that *S. boulardii* may reduce the UC carcinogenesis by inhibiting the functions of TNF-α and IL-6.

### S. Boulardii treatment alters the composition of both fecal and mucosal microbiota in AOM/DSS treated mice

**Fig. 9:**
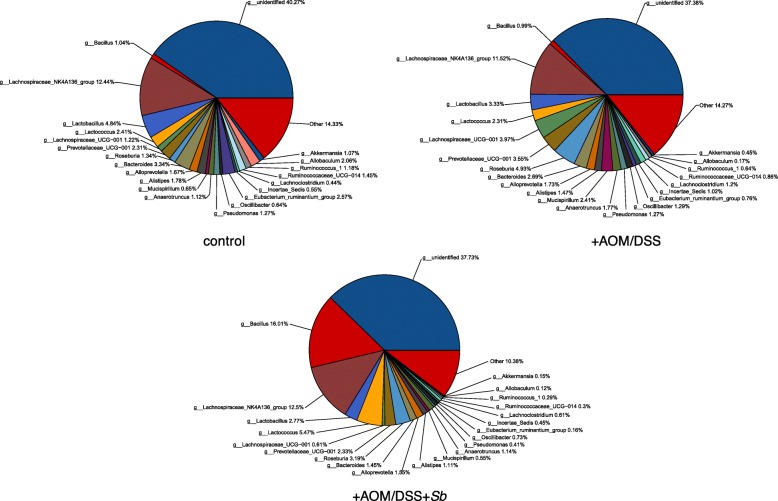
Fecal microbiota comparison among the control, the AOM/DSS treated group (+AOM/DSS), and the AOM/DSS plus *S. boulardii* treated group (+AOM/DSS + *Sb*)

Host microbiota plays an important role in the pathogenesis of UC [9]. To investigate if AOM/DSS and *S. boulardii* treatment affected the microbiota of the mice, we compared the fecal and mucosal microbiota of the control, the AOM/DSS treated, and the AOM/DSS + *Sb* treated groups. The major compositional changes between the control and the AOM/DSS treated groups in fecal microbiota were characterized by a significant decrease in Lactobacillus and significant increases in *Oscillibacter* and *Lachnoclostridium* (Fig. 9; Table 1). Notably, the increases in *Oscillibacter* and *Lachnoclostridium* were significantly recovered by *S. boulardii* treatment (Table 1). Compared to the AOM/DSS treated group, *S. boulardii* treatment also caused significant increases in *Bacillus* and *Lactococcus* but decreases in *Bacteroides* and *Pseudomonas*. No difference in microbiota composition was observed among the three groups before the treatment. Additionally, the diversity of microbiota remained similar before and after the treatment.

**Table 1:** Comparison of fecal microbiota

Genus	Control (%)	+AOM/DSS (%)	+AOM/DSS + *Sb* (%)
Lactobacillus	4.84	3.33*	2.77
Oscillibacter	0.64	1.29*	0.73^#^
Lachnoclostridium	0.44	1.2*	0.61^#^
Bacillus	1.04	0.99	16.01^#^
Lactococcus	2.41	2.31	5.47^#^
Bacteroides	3.34	2.69	1.45^#^
Pseudomonas	1.27	1.27	0.41^#^

*: Statistically significant (*p* < 0.05) between the AOM/DSS and the control groups;

#: Statistically significant (*p* < 0.05) between the AOM/DSS and the AOM/DSS + *Sb* groups

**Fig. 10:**
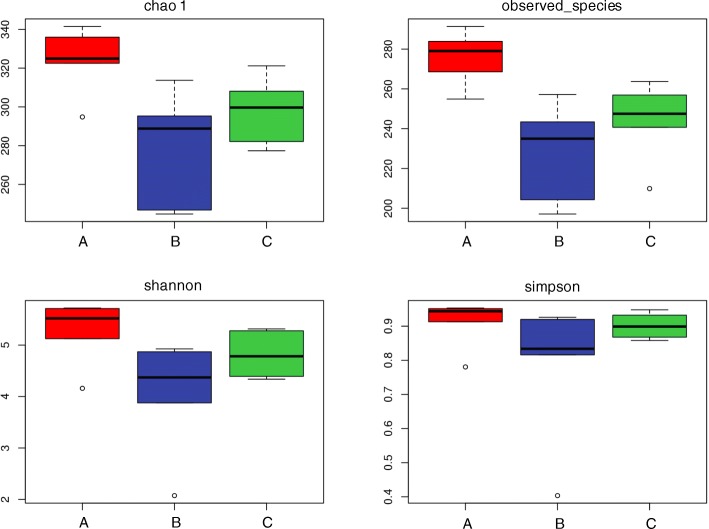
Diversity analysis of the mucosal microbiota among the AOM/DSS plus *S. boulardii* treated group (A), the AOM/DSS treated group (B) and the control (C). observed_species means the number of OTU

For the mucosal microbiota, diversity analysis using Chao 1, observed species indexes, PD_whole_tree and Shannon indexes showed that *S. boulardii* treatment resulted in a significant increase in diversity compared to the AOM/DSS treated group (*p* < 0.05), while no significant differences in community diversity were observed between the control and the AOM/DSS treated group (Fig. 10).

Anosim analysis showed a distinct shift in the microbiota composition in the *S. boulardii* treated group compared to the AOM/DSS treated group (R > 0, *p* < 0.05).

**Fig. 11:**
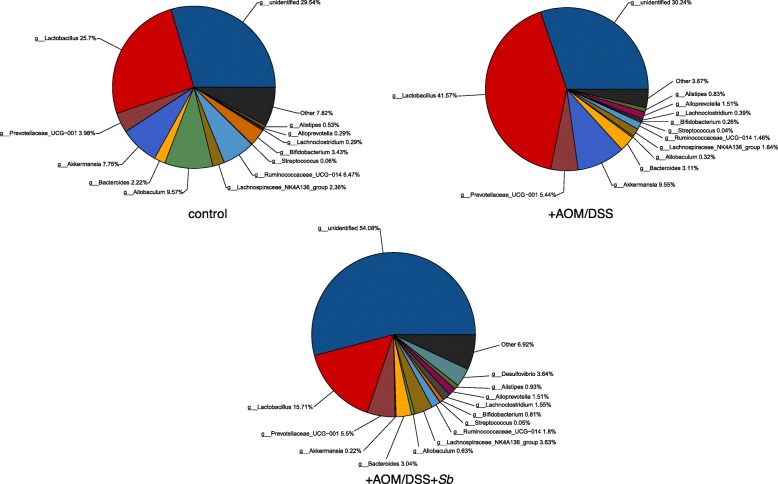
Mucosal microbiota comparison among the control, the AOM/DSS treated group (+AOM/DSS) and the AOM/DSS plus *S. boulardii* treated group (+AOM/DSS + *Sb*)

About 30% of the bacteria were unidentified in the control and AOM/DSS treated groups, while 54% of the bacteria were unidentified in the +AOM/DSS + *Sb* treated group (Fig. 11). AOM/DSS treatment also caused a significant decrease in the genus *UCG-014 of Ruminococcaceae*, but a significant increase in *Alloprevotella*. The genera *Lachnospiraceae_NK4A136* and *Lachnoclostridium* were enriched following *S. boulardii* treatment (Table 2).

**Table 2:** Comparison of mucosal microbiota

Genus	Control (%)	+AOM/DSS (%)	+AOM/DSS + *Sb* (%)
Alloprevotella	0.29	1.51*	1.51
Ruminococcaceae_UCG-014	6.47	1.46*	1.80
Bifidobacterium	3.43	0.26*	0.81
Lachnospiraceae_NK4A136	2.36	1.64	3.63^#^
Lachnoclostridium	0.29	0.39	1.55^#^

*: Statistically significant (*p* < 0.05) between the AOM/DSS and the control groups;

#: Statistically significant (*p* < 0.05) between the AOM/DSS and the AOM/DSS + *Sb* groups

## Discussion

UC carcinogenesis is a multistep process during which the epithelial cells in the colon undergo inflammation-dysplasia-carcinoma [12]. Chronic inflammation is a key factor of carcinogenesis in UC patients. Tumor necrosis factor alpha (TNF-α) and interleukin 6 (IL-6) are both pro-inflammatory cytokines that play an integral role in the pathogenesis of UC carcinogenesis. Studies have shown increased levels of TNF-α and IL-6 in UC patients [13]. In this study, we found that TNF-α and IL-6 levels in colon tissues were significantly higher in the AOM/DSS treated mice than in the control group. The AOM/DSS treated mice developed multiple colorectal tumors in the colon tissues, suggesting that TNF-α and IL-6 may be associated with tumor formation.

Studies have showed that TNF-α and IL-6 played important roles in UC carcinogenesis. TNF- α is the key participator of UC carcinogenesis. One study showed that in a UC carcinogenesis model, the tumor formation rate and tumor size in mice lacking TNF-α receptor P55 was significantly lower than in wild-type mice [14]. IL-6 is also an important inflammatory factor participating in UC carcinogenesis. Previous study found that in a UC carcinogenesis mice model, the tumor load of IL-6^−/−^ mice significantly declined than in the wild-type mice [15].

Studies have shown that probiotics have a therapeutic effect on the control of inflammation and maintenance of UC carcinogenesis remission [16]. For example, the probiotic *S. boulardii* has been shown to inhibit TNF-α and IL-6 levels as well as other pro-inflammatory cytokines, such as IL-1β and IL-8 R[17]. It has also been reported that *S. boulardii* can inhibit tumor formation in mice [18]. Consistent with these reports, the results presented herein demonstrated that *S. boulardii* treatment significantly reduced both TNF-α and IL-6 levels in AOM/DSS treated mice. Moreover, it also reduced tumor incidence as well as the tumor load (Fig. 3a). In vitro, we further demonstrated that *S. boulardii* treatment lowered the activities of TNF-α and IL-6, suggesting two possible mechanisms for *S. boulardii* UC carcinogenesis inhibition: one, to inhibit the overproduction of pro-inflammatory cytokines; and two, to block the pro-carcinogenic functions of TNF-α and IL-6. Inhibition of TNF-α and IL-6 likely affects the Wnt signaling pathway, which is integral to UC carcinogenesis [19]. One limitation of our in vitro experiment is the possible pH changes during the co-culture, which could potentially affect the cytokine secretion. However, a dramatic pH change is not highly anticipated due to the relative short co-culture time (4 h) and relative low MOI (~ 1). Microscopic examination (Fig. 4) also suggested that the addition of *S. boulardii* did not affect the cell status. Nevertheless, the pH should be measured in future experiments before and after the experiment to rule out the possible pH effect on cytokine secretion.

Intestinal microbiota plays an important role in human health. It contributes to metabolic, nutritional, physiological, and immunological processes in the human body [20]. It also participates in the defense against pathogens by producing antimicrobial compounds or through colonization resistance. To better understand the possible roles of intestinal microbiota in UC carcinogenesis, we examined changes in intestinal microbiota in mice that were treated with the oncogenic AOM/DSS, with and without probiotic *S. boulardii* treatment, and compared with healthy controls. For the fecal microbiota, we found that compared to the healthy control group, the AOM/DSS treated mice possessed less *Lactobacillus* but more *Oscillibacter* and *Lachnoclostridium*. Studies have shown that *Lactobacillus bulgaricus* can reduce colitis [21] and *Lactobacillus rhamnosus*can effectively maintain UC remission [22]. On the other hand, it has been reported that *Oscillibacter* is a potential opportunistic pathogen that is part of the human intestinal microbiota [23]. Therefore, we believe that dysbiosis, a maladaptation of the microbiota where beneficial bacteria are reduced and harmful bacteria are increased, contributes to the process of UC carcinogenesis. This also explains why the microbiota biodiversity was not significantly different between the AOM/DSS treated group and the control group.

Previous studies have shown that probiotic supplements can balance the intestinal microbiota of UC patients [16]. They are known to promote intestinal homeostasis by blocking pathogenic bacteria, increasing epithelial barrier integrity, stimulating innate immune responses, and balancing the production of inflammatory cytokines [24]. In our current study, we found that the percentage contribution of *Bacillus* and *Lactococcu*s increased, while *Bacteroides, Oscillibacter, Lachnoclostridium,* and *Pseudomonas* numbers decreased in the fecal microbiota following *S. boulardii* treatment. Several species of *Bacillus* and *Lactococcus* have been widely used as probiotics. *Bacillus subtilis*, for example, can significantly reduce DSS-induced colonic mucosal injury and inflammatory factor levels, and improve short-chain fatty acid levels [25]. *Lactococcuslactis* also assumes a protective role in DSS-induced colitis model mice [26]. *Bacteroides* and *Pseudomonas* on the other hand, can induce colitis [27, 28]. These results suggest that *S. boulardii* supplementation, following the onset of AOM/DSS-induced colitis, promotes the development of a healthier gastrointestinal microbiota that helps reduce the UC carcinogenesis induced by AOM/DSS.

In mucosal microbiota, we found that the *Ruminococcaceae genus UCG-014* and *Bifidobacterium* decreased, while *Alloprevotella* had increased in the AOM/DSS treated group, when compared with the control group. Several genera of *Ruminococcaceae* are able to produce acetate, which is then used by *Roseburia* to produce Butyrate. Butyrate is not only the main source of energy for intestinal epithelial cells, but also can inhibit the signaling pathways of pro-inflammatory cytokines [29]. It also enhances intestinal barrier function by increasing mucin secretion and the enhancement of tight-junctions [30]. Studies have shown that butyrate-producing bacteria (e.g., Clostridium clusters IV and XIVa) and butyrate concentration are reduced significantly in UC patients [31]. *Bifidobacterium* can produce bacteriocin and organic acids against pathogens upon intestinal mucosal invasion [32]. In terms of the effect of probiotic supplementation on mucosal microbiota, we found that the *NK4A136 genus* of *Lachnospiraceae* and *Lachnoclostridium* increased after treatment with *S. boulardii*. Many genera of *Lachnospiraceae* are capable of producing butyrate [33]. These results further point to the beneficial influence of *S. boulardii* in reducing UC carcinogenesis.

## Conclusion

In summary, this study demonstrated that *S. boulardii* could effectively reduce UC carcinogenesis in AOM/DSS treated model mice. This beneficial effect is likely due to multiple reasons, such as reducing the overproduction of the pro-inflammatory cytokines TNF-α and IL-6, the blockage of TNF-α and IL-6 pro-carcinogenic functions, and the rebalance of intestinal microbiota composition. Our study suggests that *S. boulardii* may be a potential therapeutic agent for UC carcinogenesis prevention and treatment.

## Data Availability

The datasets used and analyzed during the current study are available from the corresponding author on reasonable request.

## Abbreviations

AOM: Azoxymethane
CFU: colony forming unit
DMEM-H: Dulbecco’s modified eagle medium-high glucose
DSS: Dextran sulfate sodium
ELISA: Enzyme-linked immunosorbent assay
FBS: Fetal bovine serum
HE: Hematoxylin-eosin
IL-6: Interleukin 6
IMDM: Iscove’s modified Dulbecco’s medium
MEM: Minimum Eagle’s medium
MOI: Multiplicity of infection
NEAA: Non-essential amino acids
OTUs: Operational taxonomic units
PBS: Phosphate-buffered saline
TNF-α: Tumor necrosis factor alpha
UC: Ulcerative colitis.
